# Thermal Protection Performance of Biomimetic Flexible Skin for Deformable High-Speed Vehicles (DHSV-bio-FS) under Uniaxial Tensile Strain

**DOI:** 10.34133/research.0394

**Published:** 2024-06-05

**Authors:** Chao Yuan, Xiaozhou Lü, Weimin Bao

**Affiliations:** School of Aerospace Science and Technology, Xidian University, Xi’an 710071, China.

## Abstract

Vehicle skin is the key component in maintaining the aerodynamic shape of the vehicle. A deformable high-speed vehicle needs to adjust its shape in real time to realize optimum aerodynamic efficiency and to withstand extreme heat flow induced by high-speed flight, which requires the skin to possess large strain and high-temperature resistance. Traditional vehicle skin cannot satisfy both of the requirements. Biomimetic flexible skin for deformable high-speed vehicles (DHSV-bio-FS) combines flexible material fabrication with transpiration cooling technology, which can simulate human skin sweat cooling, and has the characteristics of large strain and high-temperature resistance. The thermal protection performance of the prepared prototype of DHSV-bio-FS was evaluated by simulation and wind tunnel experiments at 40% tensile strain with liquid water as coolant. Simulation results suggest that the surface temperature of the DHSV-bio-FS at 40% tensile strain is consistent with the temperature of the coolant (350 K) in a 3,000 K high-temperature gas environment. In addition, the prepared prototype DHSV-bio-FS survived for 1,200 s in a high-temperature gas environment of 200 kW/m^2^ in wind tunnel experiments. This paper verifies the reliability of DHSV-bio-FS in a high-temperature gas environment and can be deployed in applications of flexible skin for deformable high-speed vehicles (DHSV-FS).

## Introduction

During the flight of a vehicle, it undergoes diverse stages such as take-off, climb, cruise, landing, and touchdown. Traditional non-deformable high-speed vehicles are designed and optimized for their main flight tasks only; they are incapable of maintaining their best performance during all flight stages [1,2]. A deformable high-speed vehicle can actively adjust its fuselage configuration (outer shape) in real time according to different flight stages and environments to achieve the optimal performance [3–5]. Combining the advantages of deformability and high speed, a deformable high-speed vehicle can realize efficient and high-speed long-distance flight, thereby greatly reducing its flight time, which has great scientific research value and application prospect in the fields of intercontinental and space-shuttle transportation [6]. However, deformable high-speed vehicles have not been widely used due to the lack of suitable flexible skin. As an important component of the deformable high-speed vehicle [7,8], the flexible skin is the foundation for developing deformable high-speed vehicles. The deformability of the flexible skin is the prerequisite for supporting deformable flight of the vehicle. In addition, the flexible skin also requires the characteristics of high-temperature resistance under extreme heat flow on the surface of a high-speed vehicle due to high-speed flight [9–11]. In order to mitigate heat accumulation on the deformable surface of a high-speed vehicle, the flexible skin must be continuous [12]. Therefore, the ideal flexible skin for deformable high-speed vehicles must possess the characteristics of large strain, high-temperature resistance, and continuous surface. Unfortunately, these characteristics are contradictory, which limits the development of deformable high-speed vehicles.

The vehicle skin has received extensive research. In order to design a more flexible and efficient vehicle, some scholars proposed the concept of a deformable wing based on large strain characteristic of flexible skin, and applied it to the field of deformable vehicles [13–20]. Zhou et al. [13] from Zhejiang University designed a tensegrity-based morphing wing by embedding the tensegrity structure into the pre-stretched latex flexible skin. The skin primarily utilizes the elastic strain of the polymer material, which can be deformed under the action of internal driver. Sun et al. [16] from Harbin Institute of Technology reported the concept of morphing wingtip based on actively inflatable honeycomb and shape memory polymer composite (SMPC) skin. The skin is made of styryl SMPC. The out-of-plane stiffness of the wingtip was adjusted by controlling the temperature of the morphing wingtip, and the bending deformation of the wingtip was realized by placing an actively inflatable honeycomb structure inside. Kölbl and Ermanni [18] from Eidgenössische Technische Hochschule Zürich (ETH Zurich) proposed a biaxially anisotropic deformable skin composed of a poly-formaldehyde copolymer (POM-C) small unit, in which each unit is connected by a direct bond. By changing the space between these units, the stretching and bending deformation can be achieved. These researches have applied flexible skin to the field of low-speed deformable vehicles, which greatly enriched the design methodology of deformable vehicles while expanding the application field of flexible skin. Unfortunately, these researches still cannot solve the contradiction between the characteristics of DHSV-FS of large strain and high-temperature resistance. These flexible skins are unable to work stably under extreme thermal environments on the surface of a deformable high-speed vehicle because they abandon high-temperature resistance. In addition, they cannot be directly applied to the area of a deformable high-speed vehicle. Meanwhile, some scholars focused on breaking the speed limit of high-speed vehicles. The high-speed vehicle is subjected to intense aerodynamic heating during high-speed flight [9], and the extreme heat flow generated by aerodynamic heating will severely damage the skin of a high-speed vehicle or even destroy the equipment inside the vehicle. Therefore, protecting the skin against extreme heat flow has become one of the key problems to break through the speed limit of a high-speed vehicle. They applied active thermal protection technology represented by transpiration cooling technology to the design of vehicle skin [21–27], and developed rigid vehicle skin with active thermal protection function. Van Foreest et al. [21] from the German Aerospace Centre cooled the vehicle nose cone by using liquid water in 2009. The experimental result revealed the mechanism of transpiration cooling and the influence of liquid phase transition and gas film insolation on the cooling effect. Huang et al. [24] from Tsinghua University designed a rigid skin with tree-like microchannels. The microchannels inside the skin can produce capillary effect, by which the skin possesses the characteristics of self-pumping coolant. These active thermal protection rigid skins can effectively reduce the skin surface temperature and thus work stably under extreme thermal environments. The above researches have effectively solved the “aerodynamic heat” problem of high-speed vehicles and promoted their technical application. The fabrication of these active thermal protection rigid skins generally adopts porous material as the base [28], which is made of high-temperature resistant materials such as alloy or ceramic [29–31]. These materials tend to be rigid, sacrificing the stretchability of flexible materials. These rigid materials usually sacrifice the large strain of flexible materials. The aerodynamic shape of the high-speed vehicle developed based on the active thermal protection rigid skin is often immutable, sacrificing the flexibility and high maneuverability, which restricts the application of high-speed vehicles.

The development of the ideal DHSV-FS can promote the development of a deformable high-speed vehicle with optimal aerodynamic efficiency and high speed. However, the methods that aim at solving the contradiction between the characteristics of large strain and high-temperature resistance properties of DHSV-FS still confront a great challenge. To overcome this challenge, Su et al. [32] from Xi’an Jiaotong University developed a highly stretchable, crack-insensitive and compressible ceramic aerogel. The aerogel can be stretched up to 20% and exhibits excellent thermal stability at a high temperature up to 1,500 K. In addition, in our previous research, we developed a deformable biomimetic flexible skin for high-speed vehicles (DHSV-bio-FS) with large strain and high-temperature resistance [33]. The skin can be bent 180° and survive for 1,200 s under a high-temperature gas environment with 200 kW/m^2^ heat flow. These research findings provide technical support for the development of DHSV-FS. Compared with the aerogel proposed by Lei et al., the tensile strain of our proposed DHSV-bio-FS proposed can reach 60%, which greatly increases the deformation range of the vehicle. However, the influence of tensile strain on the thermal protection performance of DHSV-bio-FS was not verified. The result of the research does not indicate that the DHSV-bio-FS possesses the advantages of both large strain and high-temperature resistance.

Based on the above analysis, this paper focuses on the influence of tensile strain on the thermal protection performance of DHSV-bio-FS. Therefore, we prepared a prototype of DHSV-bio-FS and systematically evaluated the thermal protection performance of DHSV-bio-FS under different tensile strain by combining the methods of numerical simulation and wind tunnel experiments. The simulation and experimental results have verified the cooling mechanism of DHSV-bio-FS, laying theoretical foundation for the development of deformable high-speed vehicles.

## Results

### Simulation results showed that DHSV-bio-FS can survive in 3,000 K high-temperature gas

**Fig. 1.**
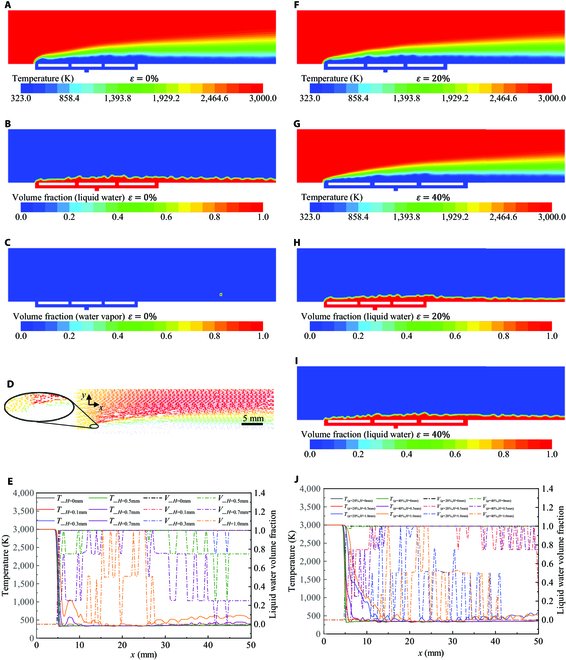
Simulation results under 3,000 K high-temperature gas conditions: (A) Diagram of temperature distribution (*ε* = 0%). (B) Diagram of liquid water distribution (*ε* = 0%). (C) Diagram of water vapor distribution (*ε* = 0%). (D) Graph of motion variation. (E) The temperature and the coolant volume fraction variation curves vary with the distance *x* at the height of 0 mm, 0.1 mm, 0.3 mm, 0.5 mm, 0.7 mm, and 1.0 mm above the DHSV-bio-FS surface. (F) Diagram of temperature distribution (*ε* = 20%). (G) Diagram of temperature distribution (*ε* = 40%). (H) Diagram of liquid water distribution (*ε* = 20%). (I) Diagram of liquid water distribution (*ε* = 40%). (J) The temperature and the coolant volume fraction variation curves vary with the distance *x* at the height of 0 mm, 0.5 mm, and 1.0 mm above the DHSV-bio-FS surface under 20% and 40% tensile strain.

As a deformable high-speed vehicle flies at velocities ranging from 5 to 25 Ma, the friction between its surface and the air generates a high-temperature field with a gaseous environment temperature exceeding 3,000 K and an aerodynamic field with a peak pressure of at least 7.5 kPa. This paper simulates the thermal protection performance of DHSV-bio-FS in a typical high-temperature gas environment of 3,000 K. The diagram of surface temperature distribution of the DHSV-bio-FS under a 3,000 K high-temperature gas environment was demonstrated (Fig. 1A). The surface temperature of the DHSV-bio-FS ranged between 323 K and 590 K, which is noticeably lower than the environment temperature (3,000 K).

A layer of water film was formed by the coolant covering the DHSV-bio-FS surface (Fig. 1B). The low-temperature region corresponds to the region covered by liquid water (Fig. 1A and B). In addition, wake occurs at the rear end of the DHSV-bio-FS surface (Fig. 1B). Only a small amount of water vapor existed in the experimental section (Fig. 1C).

The microchannels of the DHSV-bio-FS regulated the coolant to flow as evenly as possible out of the surface, then the flowing coolant accumulated on the DHSV-bio-FS surface, forming a liquid protective film (Fig. 1D). The film isolated the contact between the high-temperature gas and the DHSV-bio-FS, forcing the high-temperature gas to change its movement direction on the DHSV-bio-FS surface. At the horizontal distance (*x*) of 5 mm, it can be found that the movement directions of both high-temperature gas and liquid water change by almost 90°, at which position the largest relative movement and the most intensive collision happens between high-temperature gas and liquid water.

In order to investigate the surface temperature variation of the DHSV-bio-FS, the variation curves of the coolant temperature (*T*) and of the volume fraction (*V*) at different heights (*H*) of 0 mm, 0.1 mm, 0.3 mm, 0.5 mm, 0.7 mm, and 1.0 mm above the DHSV-bio-FS surface were demonstrated (Fig. 1E), both of which vary with *x*. When *x* = 5 mm, the temperature sharply decreases from 3,000 K to 340 K at *H* = 0 mm. Subsequently, the surface temperature of DHSV-bio-FS (*x* > 5 mm) is invariably lower than 473.15 K, which locates within the temperature range where the DHSV-bio-FS can function stably. According to the curves of the temperature variation and of the coolant distribution, it can be determined that the variation trend of the surface temperature of DHSV-bio-FS is basically consistent with that of the coolant volume fraction. Within the region where the coolant volume fraction is 1, the surface temperature of DHSV-bio-FS remains almost the same as that of the coolant. The temperature distribution curve varies most markedly at *x* = 5 mm. At *x* > 10 mm, the coolant accumulates, strengthening the thermal protection performance of DHSV-bio-FS. The temperature variation at this position gradually stabilizes, and the temperature remains at approximate 350 K.

As *H* increases, the surface temperature of DHSV-bio-FS increases accordingly. When *H* = 0.5 mm, the curve of the coolant volume fraction of the DHSV-bio-FS surface varies drastically, but the coolant volume fraction is still more than 0.8, for which reason the surface temperature of DHSV-bio-FS still remains lower than 473.15 K. When *H* = 0.7 mm, insufficient coolant can be observed noticeably in some working areas, and the temperature of these areas exceeds 473.15 K, which is beyond the temperature-resistance range of flexible materials.

### High tensile strain reduced thermal protection performance of DHSV-bio-FS

In order to investigate the thermal protection performance of the as-prepared DHSV-bio-FS under different tensile strains, the tensile strain (*ε*) of the DHSV-bio-FS was set to 20% and 40% respectively to conduct the corresponding simulations. The diagram of temperature distribution under 20% and 40% tensile strain of DHSV-bio-FS are demonstrated respectively (Fig. 1F and G). The temperature distribution trends of the DHSV-bio-FS surface are consistent under different tensile strains (Fig. 1A, F, and G). The distribution area of the coolant is consistent with that of the low-temperature area (Fig. 1F and H and Fig. 1G and I). At *ε* = 40%, the surface temperature of the DHSV-bio-FS noticeably decreases to the temperature range from 323 K to 456.85 K.

Comparing various heights (*H* = 0 mm, *H* = 0.3 mm, *H* = 0.5 mm, *H* = 0.7 mm, and *H* = 1 mm) on the surface of DHSV-bio-FS under the condition of *ε* = 0%, this paper focuses on the temperature variations at the typical heights of *H* = 0 mm, *H* = 0.5 mm, and *H* = 1 mm at *ε* = 20% and *ε* = 40%. The curves of the temperature and of the coolant volume fraction vary with the distance *x* at *H* = 0 mm, 0.5 mm, and 1.0 mm above the surface of the DHSV-bio-FS under *ε* = 20% and 40% (Fig. 1J). The temperature distribution curves exhibit the same variation trend under different tensile strains. The thermal protection performance of the DHSV-bio-FS decreases as the height increases. At *H* = 0 mm above the skin surface, the temperature is consistent with that of the coolant. At *H* = 0.5 mm and *x* = 5 to 6.6 mm, the surface temperature of the DHSV-bio-FS with *ε* = 40% drops from 2,100 K to 500 K, exceeding the upper limit of high-temperature resistance of the prepared DHSV-bio-FS (480 K). The cooling film upstream at *ε* = 20% is thicker than that at *ε* = 40%. This is because when the tensile strain increases, the hole spacing of water outlets is stretched and increased from 7.44 mm to 8.68 mm, by which the forming of the protective film by a single outlet hole is therefore encumbered. However, the thickness variation of the cooling film downstream is not apparent.

### The DHSV-bio-FS prototype survived in a high-temperature gas environment under tensile strain

**Fig. 2.**
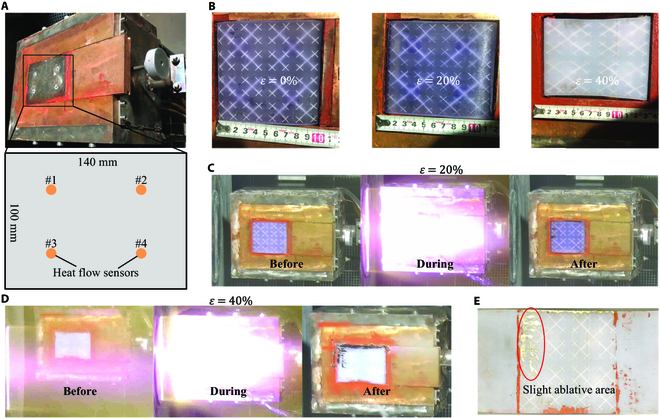
(A) Heat flow calibration. (B) Different tensile strains of DHSV-bio-FS. (C) Thermal experiments of DHSV-bio-FS under 20% tensile strain. (D) Thermal experiments of DHSV-bio-FS under 40% tensile strain. (E) Prototype after experiment.

According to the simulation results, the thermal experiments were conducted under 20% and 40% tensile strain of the prepared DHSV-bio-FS in a high-temperature gas environment with a heat flux (*q*) of 200 kW/m^2^. The heat flow was calibrated for 40% tensile strain (Fig. 2A). In order to verify the consistency of the heat flow, calibration was conducted 3 times, to which the heat flow values of the 4 calibration components on the skin surface are shown in Table S1.

During the experiment, the pipes on the leftmost side of the DHSV-bio-FS were aligned flush with the left side of the fixture, while the pipes on the rightmost side were aligned flush with the right side of the fixture. The test area measured 93.75 mm × 100 mm to prevent damage to the flexible skin upstream caused by non-existent pipes. The DHSV-bio-FS was stretched to 20% and 40% tensile strain and deployed on the fixture respectively (Fig. 2B). The 20% and 40% experimental specimens are 2 skins prepared from different batches, and the color difference does not affect the internal structure and tensile properties. The test progress of the DHSV-bio-FS under 20% and 40% strain was shown (Fig. 2C and D). It can be confirmed that the prepared DHSV-bio-FS remained basically intact after the experiment. In addition, liquid wake flow occurred during the experiment, which can be attributed to the joint effect of high-temperature gas and gravity on the skin.

The prepared DHSV-bio-FS can work stably for 1,200 s in high-temperature gas with *q* = 200 kW/m^2^ under the condition of *ε* = 20% and 40% (Fig. 2C and D). During the 1,200 s experiment, the DHSV-bio-FS thermal protection performance has become stable, which is enough to extend the working time in high-temperature gas environments while maintaining a steady coolant supply. On the DHSV-bio-FS surface, a slight ablative area occurred (Fig. 2E), which was shallow and did not go deeper inside. Throughout the whole experiment, the prepared DHSV-bio-FS was not torn.

### The DHSV-bio-FS prototype maintained tensile properties

**Fig. 3.**
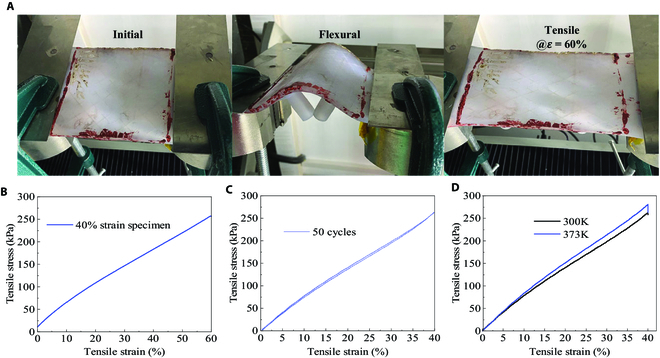
(A) Strain properties test. (B) Stress–strain curve. (C) Fifty cycles of stretching experiments. (D) Stress–strain curves at 300 K and 373 K.

This paper conducted tensile property testing experiments at room temperature (300 K) on the 40% stretched DHSV-bio-FS specimen previously used in the thermal experiment. The experiments investigate the effect of thermal experiments on the mechanical properties of DHSV-bio-FS stretched to maximum strain. Forty percent stretched DHSV-bio-FS specimen exhibited excellent strain properties after the thermal experiment (Fig. 3A). The stress–strain curve of the DHSV-bio-FS specimen was measured at 60% tensile strain at room temperature (Fig. 3B), and the Young’s modulus of the DHSV-bio-FS specimen was calculated to be 542 kPa. Fifty cycles of stretching experiments were conducted on the DHSV-bio-FS specimen at room temperature (Fig. 3C), and no irreversible deformation was observed. The stress–strain curves of DHSV-bio-FS were also measured at 300 K and 373 K (Fig. 3D). It was observed that the Young’s modulus of DHSV-bio-FS increased at 373 K.

## Discussion

Flexible skins with large strain and high-temperature resistance are the key components of deformable high-speed vehicles to modify aerodynamic shapes and withstand aerodynamic heating. Flexible skins with large strain can be applied to a variety of complex surface shapes and have attracted much attention in the field of deformable vehicles. Active thermal protection technology can reduce the effect of heat on the object and is highly flexible, which has a broad application prospect in the field of high-speed vehicles. Therefore, the combination of flexible skin technology and active thermal protection technology is one of the effective methods to develop flexible skins for deformable high-speed vehicles.

The integration of flexible skins with large strain and high-temperature resistance, as well as active thermal protection technology, is a highly effective approach for developing flexible skins for deformable high-speed vehicles. These flexible skins are crucial for altering aerodynamic shapes and withstanding aerodynamic heating. They can adapt to complex surface shapes and have garnered significant attention in the field of deformable vehicles. Additionally, active thermal protection technology offers great potential for reducing the impact of heat on objects and is highly flexible, making it a promising solution for high-speed vehicles.

This paper presents a DHSV-bio-FS, and conducts simulation analysis and experimental verification of its thermal protection performance under uniaxial tensile strain. The thermal protection process of DHSV-bio-FS involves 3 stages: thermal isolation by cooling film and heat absorption of the coolant and of its phase change. During the above process, the liquid coolant is sprayed out of the skin surface to form a continuous liquid cooling film, by which the DHSV-bio-FS is isolated from the high-temperature gas and prevented from directly participating in heat exchange. The cooling film exchanges heat with the high-temperature gas, during which the coolant temperature rises and absorbs some heat. Then, the temperature reaches the phase transition threshold of the coolant, and the coolant continues to absorb heat to complete the phase transition process. Owing to the high latent heat of vaporization, the surface temperature of DHSV-bio-FS is effectively reduced. The coolant moves backward under the shear force of high-temperature gas, and then gathers with the spilt coolant at the rear end to form a thicker cooling film, which strengthens the thermal protection performance of the DHSV-bio-FS downstream. As the *ε* increases, the hole spacing increases accordingly, by which the forming of the protective film by a single outlet hole is therefore encumbered. Despite the above situation, the DHSV-bio-FS still works stably under a 3,000 K high-temperature gas environment at *ε* = 40%.

The experiment results suggest that the DHSV-bio-FS can survive for 1,200 s in a high-temperature gas environment with a heat flux of 200 kW/m^2 ^under 20% and 40% tensile strain. After the experiment, the DHSV-bio-FS still exhibits bending performance while maintaining 60% tensile performance and excellent tear resistance. The experiment results are consistent with the simulation results.

In conclusion, this paper proposes a biomimetic flexible skin for deformable high-speed vehicles (DHSV-bio-FS). Compared with traditional flexible skins, the DHSV-bio-FS possesses both large strain and high-temperature resistance, enabling it to work stably in the extreme thermal environment on the surface of the deformable high-speed vehicle. The development and validation of this flexible skin provides a method for the development of flexible skins for deformable high-speed vehicles.

## Materials and Methods

### Working mechanism of DHSV-bio-FS

The DHSV-bio-FS was designed by imitating the process of human skin sweating and cooling [34]. When the amount of heat produced by human exercises increases skin temperature, the nerve receives the signal and stimulates the sudoriferous glands to secrete sweat. Then, the sweat flows out of the pores, heating up and changing phase on human skin surface. During the above process, a great amount of heat dissipates to maintain the temperature of human skin within an appropriate range [35]. In order to simulate this process, the DHSV-bio-FS made of flexible materials was used to mimic human skin inside which the microchannels simulating the capillary pipe were prepared, and pumps were used to simulate the sudoriferous glands. Under general circumstances, liquid water is a preferred coolant due to its high specific heat and latent heat of phase transition [36,37].

When the DHSV-bio-FS works, the pump outside pumps out the coolant along the microchannels inside the DHSV-bio-FS, covering its surface to form a liquid cooling film. The thermal protection of the DHSV-bio-FS consists of 3 parts [10]: heat isolation by cooling film, heat absorption by heating of coolant, and by coolant phase change (Fig. S1A). The coolant avoids the DHSV-bio-FS from being in direct contact with high-temperature gas. During the contact process between the coolant and high-temperature gas, the high-temperature gas continuously heats the coolant. When the coolant temperature reaches its phase transition threshold, the phase transition dissipates a huge amount of heat. The remaining coolant moves backward under the action of the high-temperature gas shear force, and the new low-temperature coolant flows out from the pipe to replenish the remaining coolant. In the above process, the temperature of the DHSV-bio-FS surface is consistent with that of the coolant. Since the threshold temperature of the phase transition of coolant is lower than the temperature-resisting limit of flexible materials, the DHSV-bio-FS can therefore work stably under extreme thermal environments.

### Finite element simulation settings

Ansys Fluent was used as the finite element analysis software [38]. The smallest microchannel structure in DHSV-bio-FS was selected for the simulation structure. The experimental section is set up (Fig. S1B). Both the boundary condition of the high-temperature gas inlet and the coolant inlet are set as “velocity inlet”. Both directions of the high-temperature gas and the coolant are perpendicular to the wall surface. The high-temperature gas flows in at 2 m/s and the coolant flows in at 0.1 m/s. The outlet is set as “pressure outlet”. Perform the “Volume of Fluid” model according to the coolant; select the standard “k-ε” flow model. Argon is selected as the high-temperature gas, and liquid water is selected as the coolant, to which the meshing results are shown in Fig. S1C. The maximum grid size is 0.2 mm, and the average element quality is 0.988.

### Preparation of prototype

The aim of this paper is to develop a flexible skin with large stain and high-temperature resistance. The material must have the following characteristics: elastic elongation in the range of 0% to 40% and good thermal stability at the temperature of 373 K. In this paper, polydimethylsiloxane (PDMS) (SYLGARDTM 184 from Dow Chemical Company) was used as the base material, and the DHSV-bio-FS prototype was manufactured by the lost wax method [33]. The reasons for choosing PDMS in this paper include the following: (a) PDMS has good tensile properties, which can achieve elastic strains in the range of 80%; PDMS also has good encapsulation, which can conform to the surface of the vehicle; (b) PDMS has good thermal stability, which can work steadily for a long time at a temperature of 400 K without decomposition or softening; (c) PDMS has good plasticity for making fine tubes, which is widely used in microfluidics.

The fabrication process of the DHSV-bio-FS prototype is shown in Fig. S1D: (a) The desired sacrificial mold was fabricated from paraffin wax using additive manufacturing techniques. (b) The flowable PDMS pre-solids were poured into the mold and vacuumed to remove internal air bubbles. (c) Once the PDMS had cured, the sacrificial mold was removed by 90 °C water-bath heating. (d) The DHSV-bio-FS prototype with internal microfluidic channels was obtained. The physical dimensions of the DHSV-bio-FS prototype are 200 mm × 100 mm × 5 mm, to which the experimental area is 100 mm × 100 mm in the center, and 50 mm × 100 mm margins are reserved on both sides for clamping (Fig. S1E).

The internal microchannels started with 4 main channels and each main channel branched into 4 secondary channels. Each secondary channel continued to branch until the exit holes formed a 16×16 array evenly distributed on the skin surface. The diameter of the exit holes was 0.6 mm, the distance between the holes was 6.25 mm, and the diameter ratio between 2 adjacent levels of channels was approximately 1:1.4.

### Thermal test experiment

We used the heat transfer from the high temperature gas to the DHSV-bio-FS specimen to verify the thermal protection performance of DHSV-bio-FS [24,25,39,40]. The thermal testing of the DHSV-bio-FS was conducted in a wind tunnel (Fig. S1F). The system consists of a high-temperature gas generator, a fixture, and a coolant supplement system. The high-temperature gas generator was provided by the Key Laboratory of Equipment Efficiency of Ministry of Education in Extreme Environment, Xidian University. The fixture is homemade, which is made of copper and cooled by water. The coolant supplement system includes peristaltic pump and silicone hose. Kamoer DIPump550-B403 series are selected for the peristaltic pump with a maximum flow rate of 6 ml/s.

The clamping method used for DHSV-bio-FS is shown in Fig. S1G. Two sides of DHSV-bio-FS were bent at a 90° angle and then embedded in the fixture, which was secured by 2 clamping pieces. The wind tunnel nozzle, which is a 50 mm × 250 mm rectangular nozzle, was positioned close to the fixture and parallel to the surface of the flexible skin. High-temperature gas was ejected from the nozzle at subsonic speed and covered the DHSV-bio-FS. In this paper, the prepared DHSV-bio-FS prototypes were individually stretched to 20% and 40% tensile strain and exposed to high-temperature gas. During the experiment, liquid water was used as the coolant and argon was used as the high-temperature gas.

Generally, when the body temperature rises, the nervous system receives a signal and then stimulates the sweat glands to secrete sweat, thus regulating the body temperature. In this work, we regulate the flow rate of the coolant by controlling the speed of the peristaltic pump. In order to realize the survival of DHSV-bio-FS in the high-temperature gas environment, the flow rate of the coolant was fixed within the maximum flow rate of the pump, which is 6 ml/s.

Since it is difficult to measure the temperature of the high-temperature gas directly, in this paper, we therefore measured the heat flux on the surface of DHSV-bio-FS. The surface heat flux of DHSV-bio-FS was measured using a heat flux sensor. The heat flux sensor consists of a copper plunger and a K-type thermocouple wire. The measuring principle is expressed as

$$q=\frac{dQ}{s\cdot \mathrm{dt}}=\frac{\mathrm{cm}\cdot dT\;\;}{s\cdot \mathrm{dt}}=\frac{\text{cρsl}\cdot dT}{s\cdot \mathrm{dt}}=\mathrm{c\rho l}\frac{\mathrm{dT}}{\mathrm{dt}}$$ (1)

where *Q* is the total heat received by the copper plunger, *c* is the specific heat capacity of the plunger, *ρ* is the density of the plunger, *T* is the temperature, *t* is the time, *l* is the length of the plunger, and *s* is the cross-sectional area of the plunger. The heat flux of high-temperature gas can be obtained by measuring the rate of temperature rise on the back of the plunger.

### Tensile property experiment

The tensile properties of the prepared DHSV-bio-FS were tested using an electronic universal material testing machine (Fig. S1F). The model of the machine is Jiangsu Tianyuan TY-8000 series. One end of the DHSV-bio-FS is fixed and the other end is fixed to the moving beam of the machine. The machine moves the beam up and down to stretch the skin. The tensile displacement and stress of the DHSV-bio-FS are recorded by sensors.

The tensile strain of DHSV-bio-FS *ε* is expressed as

$$\varepsilon =∆l/l_{0},$$ (2)

where ∆*l* is the variation in length and *l*_0_ is the original length.

The Young’s modulus *E* was used to characterize the deformation resistance of DHSV-bio-FS, which can be calculated by

$$E=F/\left(A\cdot \varepsilon \right),$$ (3)

where *F* is the force on the DHSV-bio-FS and *A* is the cross-sectional area of the DHSV-bio-FS.

## Data Availability

The data that support the findings of this work are available from the corresponding author upon reasonable request.
