# Supplementary material for: Thermal Protection Performance of Biomimetic Flexible Skin for Deformable High-Speed Vehicles (DHSV-bio-FS) under Uniaxial Tensile Strain
Source: Research (Wash D C). 2024 Jun 5;7:0394. doi: 10.34133/research.0394 (PMC11152053; doi:10.34133/research.0394)
Supplement: Supplementary 1 — Fig. S1 Table S1 [file research.0394.f1.docx]

Supplementary Materials

**Title**

**Thermal Protection Performance of Biomimetic Flexible Skin for Deformable High-speed Vehicle (DHSV-bio-FS) under Uniaxial Tensile Strain**

**Authors**

Chao Yuan ^1^, Xiaozhou Lü ^1*^, Weimin Bao ^1^

**Affiliations**

^1^ School of Aerospace Science & Technology, Xidian University, Xi’an, 710071, China

*Address correspondence to: Lü Xiaozhou; [xzlu@xidian.edu.cn](mailto:xzlu@xidian.edu.cn)

**Fig. S1.** (A) Thermal protection mechanism of DHSV-bio-FS; (B) Experimental setup; (C) Mesh division result; (D) DHSV-bio-FS preparation process; (E) DHSV-bio-FS prototype; (F) Experimental device for extreme heat experiment; (G) DHSV-bio-FS clamping method; (H) Experiment device for tensile property test.

**Table S1** Heat flow calibration results

| No. | Result1  (kW/m^2^) | Result2  (kW/m^2^) | Result3  (kW/m^2^) |
| --- | --- | --- | --- |
| #1 | 172 | 147 | 156 |
| #2 | 67 | 52 | 66 |
| #3 | 206 | 201 | 194 |
| *#4* | 127 | 123 | 115 |
